# Inter-centre heterogeneity, temporal evolution, and factors associated with treatment selection and outcomes in chronic inflammatory demyelinating polyradiculoneuropathy: a multicentre, combined prospective and retrospective observational study

**DOI:** 10.1016/j.eclinm.2026.104031

**Published:** 2026-06-23

**Authors:** Alberto De Lorenzo, Maria Margherita Sechi, Fiore Manganelli, Dario Cocito, Chiara Briani, Yuri Falzone, Anna Mazzeo, Angelo Schenone, Vincenzo Di Stefano, Giuseppe Cosentino, Girolama Alessandra Marfia, Luana Benedetti, Marinella Carpo, Massimiliano Filosto, Luca Leonardi, Marco Luigetti, Sabrina Matà, Giuseppe Piscosquito, Tiziana Rosso, Marta Lucchetta, Gabriele Siciliano, Giuseppe Lauria Pinter, Maurizio Inghilleri, Teresa Cantisani, Francesca Notturno, Dario Ricciardi, Francesco Habetswallner, Elisa Vegezzi, Elisa Bianchi, Pietro Emiliano Doneddu, Eduardo Nobile-Orazio

**Affiliations:** a Neuromuscular and Neuroimmunology Unit, IRCCS Humanitas Research Hospital, Via Manzoni 56, 20089, Rozzano, Milan, Italy; b Child Neurology and Neuropsychiatry Unit, Department of Medicine, Surgery and Pharmacy, University Hospital of Sassari, Sassari, Italy; c Department of Neuroscience, Reproductive Sciences and Odontostomatology, University of Naples ‘Federico II’, Napoli, Italy; d Department of Clinical and Biological Sciences, University of Turin, Torino, Italy; e Neurology Unit, Department of Neuroscience, University of Padua, Padova, Italy; f Division of Neuroscience, Department of Neurology, Institute of Experimental Neurology (INSPE), San Raffaele Scientific Institute, Milano, Italy; g Department of Clinical and Experimental Medicine, Unit of Neurology, University of Messina, Messina, Italy; h Neurology Clinic, IRCCS Ospedale Policlinico San Martino Genova, Genova, Italy; i Department of Biomedicine, Neuroscience, and advanced Diagnostic (BiND), University of Palermo, Palermo, Italy; j Department of Brain and Behavioral Sciences, University of Pavia, Pavia, Italy; k Dysimmune Neuropathies Unit, Department of Systems Medicine, Tor Vergata University of Rome, Roma, Italy; l Department of Neurology, ASST Bergamo Ovest-Ospedale Treviglio, Treviglio, Italy; m Department of Clinical and Experimental Sciences, University of Brescia; NeMO-Brescia Clinical Center for Neuromuscular Diseases, Brescia, Italy; n Neuromuscular and Rare Disease Centre, Neurology Unit, Sant'Andrea Hospital, Rome, Italy; o Neurology Department, Fondazione Policlinico Universitario Agostino Gemelli IRCCS, Università Cattolica del Sacro Cuore, Roma, Italy; p Dipartimento Neuromuscoloscheletrico e degli organi di Senso, Neurology Unit, University Hospital Careggi, Firenze, Italy; q Department of Neurology, Azienda Ospedaliera Universitaria San Giovanni di Dio e Ruggi d'Aragona, Salerno, Italy; r UOC di Neurologia, Ospedale San Bassano, Vicenza, Italy; s UOC Neurologia, Ospedale Santa Maria della Misericordia, Rovigo, Italy; t Neurology Unit, Department of Clinical and Experimental Medicine, University of Pisa, Pisa, Italy; u Unit of Neuroalgology, IRCCS Foundation ‘Carlo Besta’ Neurological Institute, Milano, Italy; v Department of Human Neurosciences, Rare Neuromuscular Diseases Centre, Sapienza University of Rome, Rome, Italy; w Servizio di Neurofisiopatologia, Azienda Ospedaliera di Perugia, Perugia, Italy; x Neurofisiopatologia, Ospedale SS Pescara, Pescara, Italy; y Clinical Neurophysiology Unit, Cardarelli Hospital, Naples, Italy; z IRCCS Mondino Foundation, Pavia, Italy; aa Istituto di Ricerche Farmacologiche Mario Negri IRCCS, Via Mario Negri 2, Milano, Italy; ab Department of Neuroscience, Rehabilitation, Ophthalmology, Genetics, Maternal and Child Health, University of Genoa and IRCCS AOU San Martino-IST, Genova, Italy; ac Department of Medical Biotechnology and Translational Medicine, Milan University, Milano, Italy; ad IRCCS Neuromed, Pozzilli, Italy; ae Department of Biomedical Sciences, Humanitas University, Via Rita Levi Montalcini 4, 20072, Pieve Emanuele, Milan, Italy

**Keywords:** Chronic inflammatory demyelinating polyradiculoneuropathy, CIDP, Real world, Guidelines, Treatment

## Abstract

**Background:**

Chronic inflammatory demyelinating polyradiculoneuropathy (CIDP) is a treatable immune-mediated neuropathy, yet real-world evidence on therapeutic strategies and their evolution over time remains limited. This study aimed to characterise prescription patterns across Italian centres, assess adherence to international guidelines, and identify clinical, structural, and temporal determinants of therapeutic decision-making.

**Methods:**

This multicentre, combined prospective and retrospective observational study used data from the Italian CIDP registry, including 653 patients from 24 tertiary centres from 2015 to 2025. Detailed treatment histories were collected for induction and maintenance regimens, including intravenous immunoglobulin (IVIg), corticosteroids, plasma exchange (PE), subcutaneous immunoglobulin (SCIg), and immunosuppressants. A structured questionnaire assessed centre-level factors influencing treatment choices.

**Findings:**

Marked inter-centre heterogeneity across all treatment options was observed (p < 0.001). Over time, IVIg and SCIg use increased, while corticosteroids, PE, and traditional immunosuppressants declined. Independent determinants of induction therapy included patient characteristics, treatment period, and treating centre. Lower IVIg induction doses and oral corticosteroids were associated with worse long-term outcomes. Over time, induction response rates, residual disability, and therapy suspension rates improved. Questionnaire data revealed that although guideline recommendations strongly influenced decisions, organisational constraints, local prescribing culture, and patient preferences independently shaped treatment allocation.

**Interpretation:**

CIDP management in routine clinical practice is highly heterogeneous and influenced by both clinical and structural factors. These findings underscore the need for standardised, evidence-based, and sustainable treatment pathways to reduce unwarranted variability and ensure equitable access to optimal care as therapeutic options continue to expand.

**Funding:**

Grant from Regione Lombardia, Italy; Grant from Ministero della Salute, Ricerca Finalizzata; Kedrion Biopharma (Italy); CSL Behring (Italy); Humanitas Research Institute (Milan, Italy); GBS-CIDP Foundation International (USA).

**Research in context**

**Evidence before this study**

A PubMed search conducted on Dec 10, 2025 using the terms “CIDP” combined with “real-world treatment”, “treatment patterns”, “treatment practice”, “therapeutic strategies”, and “treatment variability” identified a limited number of relevant publications. Only five studies addressed real-world CIDP treatment patterns, mainly based on insurance claims data or physician surveys, and documented variability between centres or countries. These studies lacked detailed phenotypic information, did not evaluate temporal trends, and did not assess centre-level structural or organisational determinants of treatment selection. No study integrated a large national CIDP registry with systematic centre questionnaires and multivariable modelling of treatment choice.

**Added value of this study**

This study provides a large real-world analysis of CIDP treatment patterns in 653 patients managed across 24 specialised centres. By integrating a national clinical registry with standardised centre-level questionnaires and multivariable modelling, we systematically examined how induction and maintenance therapies are selected in routine practice and how these choices have evolved over two decades. We show that therapeutic allocation varies widely across centres and is only partly explained by clinical characteristics. Organisational constraints, local prescribing culture, and the weight attributed to patient preferences emerged as major and independent determinants of treatment selection. Clear temporal trends were identified, including increasing use of intravenous immunoglobulins (IVIg) and rituximab, declining use of plasma exchange and traditional immunosuppressants, and the progressive adoption of subcutaneous immunoglobulins (SCIg). We also identified specific areas where practice diverges from guideline recommendations, such as reduced IVIg loading doses and combined induction regimens. Together, these findings provide new insight into how CIDP treatments are delivered in daily practice and clarify the structural drivers underlying inter-centre heterogeneity.

**Implications of all the available evidence**

Effective therapies for CIDP are available, yet their use in routine clinical practice remains highly variable and is frequently influenced by non-clinical factors. Improving adherence to guideline recommendations, ensuring appropriate dosing, and promoting more standardised maintenance strategies may enhance equity of care and clinical outcomes. The prominent role of organisational constraints and local prescribing culture suggests that system-level actions, such as strengthening guideline implementation, harmonising reimbursement frameworks, and supporting resource-limited centres, are required to reduce unwarranted variability. As new and high-cost therapies for CIDP enter clinical practice, addressing these structural determinants will be critical to ensure equitable, sustainable, and evidence-based care.

## Introduction

Chronic inflammatory demyelinating polyradiculoneuropathy (CIDP) is a rare, treatable immune-mediated neuropathy characterised by progressive or relapsing motor and sensory dysfunction due to demyelination of peripheral nerves and nerve roots.^1^ Early and effective treatment is essential to prevent irreversible axonal loss and preserve functional recovery.^2^^,^^3^ CIDP may lead to substantial and persistent disability, with marked effects on quality of life and independence,^2^ and carries a considerable economic burden, largely attributable to long-term therapy.^4^

According to the 2021 European Academy of Neurology/Peripheral Nerve Society (EAN/PNS) guideline, intravenous immunoglobulin (IVIg) or corticosteroids are strongly recommended as first-line induction treatments in typical and variant forms of CIDP, except for motor CIDP, for which IVIg is preferred.^5^ Plasma exchange (PE) is advised for non-responders.^5^ Maintenance therapy may include IVIg, subcutaneous immunoglobulin (SCIg), or corticosteroids, whereas immunosuppressants are suggested only for refractory cases or when high doses of first-line treatments are required.^5^ These recommendations are supported by randomised controlled trials and observational studies showing comparable efficacy of IVIg, corticosteroids, and PE, with IVIg offering a favourable safety profile.^5^, ^6^, ^7^, ^8^, ^9^, ^10^, ^11^ SCIg has also demonstrated efficacy as a maintenance therapy in a phase III trial.^12^ Meanwhile, several novel therapeutic classes, including B-cell–targeted agents, complement inhibitors, and neonatal Fc receptor (FcRn) antagonists, are under investigation, and the FcRn blocker efgartigimod alfa/hyaluronidase-qvfc recently obtained FDA (2024) and EMA (2025) approval.^13^^,^^14^

Despite these therapeutic advances, real-world evidence on CIDP treatment patterns remains limited. Existing studies consist mainly of small cohorts or physician surveys,^15^, ^16^, ^17^, ^18^, ^19^ and high-quality data on the determinants of treatment selection and the evolution of prescribing practices over time are lacking. Available reports suggest substantial inter-centre and international variability, influenced by healthcare system characteristics, drug availability, economic constraints, and potentially clinician discretion.^15^, ^16^, ^17^, ^18^, ^19^ The absence of validated biomarkers, limited comparative trials, and variability in guideline interpretation further contribute to uncertainty in treatment selection.^5^

**Supplementary Figure S1:**
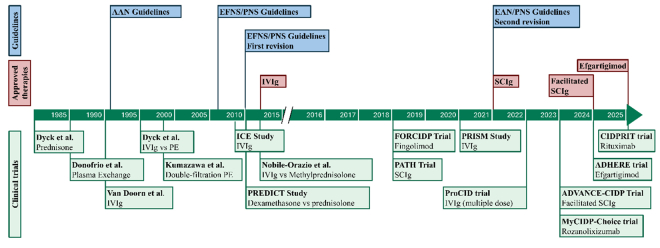
**Evolution of the therapeutic landscape of CIDP**. LEGEND: AAN, American Academy of Neurology; EAN, European Academy of Neurology; EFNS, European Federation of Neurological Societies; PNS, Peripheral Nerve Society; IVIg, intravenous immunoglobulins; PE, plasma exchange; SCIg, subcutaneous immunoglobulins.

The evolution of therapeutic patterns in routine practice, particularly in relation to guideline updates (Supplementary Figure S1), remains unclear.^5^^,^^20^, ^21^, ^22^ Understanding these dynamics is essential for optimising patient care and guiding future clinical and regulatory strategies.

This study aimed to characterise treatment patterns across Italian CIDP centres, assess adherence to international guidelines, and evaluate how therapeutic practices have changed over time. These insights are critical for informing standardised care pathways, future clinical trials, and sustainable healthcare planning.

## Methods

### Study deign and population

In 2015, we established a web-based national registry to collect data from Italian patients diagnosed with CIDP. The registry includes both retrospectively and prospectively enrolled cases. Data were initially entered by the treating neurologists into a platform developed by CINECA (Bologna, Italy) and subsequently migrated to the REDCap system. All diagnoses of typical CIDP and variants were reviewed by the coordinating centre (PED. and ENO), in agreement with the treating neurologist, and classified according to the 2010 European Federation of Neurological Societies/Peripheral Nerve Society (EFNS/PNS) diagnostic criteria, with subsequent reclassification based on the 2021 EAN/PNS criteria.^5^^,^^21^ After diagnostic revision, patients with available clinical and treatment data from centres contributing more than 10 cases were included in the analysis. Screening for IgG antibodies against nodal and paranodal proteins was performed as part of a previously conducted multicentre study within the same registry network.^23^

At enrolment, patients underwent a standardised clinical assessment, including a detailed medical history documenting symptom onset, distribution, and progression of weakness, sensory disturbances, ataxia, pain, cramps, tremor, fatigue, and cranial nerve impairment. This information was complemented by medical record review. Acute-onset CIDP (A-CIDP) was documented and defined as a rapidly progressive neuropathy, often initially diagnosed as Guillain-Barré syndrome (GBS), that progressed or relapsed beyond two months from symptom onset. Clinical evaluation comprised the Medical Research Council (MRC) sum score (12 muscles, range 0–60), the Inflammatory Neuropathy Cause and Treatment (INCAT) disability scale (range 0–10), and the Inflammatory Rasch Overall Disability Scale (I-RODS; raw score, range 1–48).^24^^,^^25^ For the present analysis, only clinical measures recorded before treatment initiation were included.

Nerve conduction studies were collected and interpreted according to local normative values, with demyelinating features defined according to the 2010 EFNS/PNS electrodiagnostic criteria.^21^

Treatment history was documented in detail, including the type of therapy, route of administration, dosage, frequency, and start and end dates. Induction therapy was defined as the initial treatment administered to induce remission; maintenance therapy referred to regimens used to sustain disease control. Treatment response was defined as a subjective improvement confirmed by an increase of at least 2 points in the MRC sum score (range 0–60) or at least 1 point in the INCAT score (range 0–10).^5^ Response data were obtained retrospectively at registry entry and prospectively during follow-up. Treatment attempts were defined as the number of different therapeutic strategies initiated before achieving clinical response. Minimal residual disability at last follow-up was defined as an INCAT score of 0–1 after at least one year of therapy. Patients who completely discontinued all immunomodulatory therapies were classified as having achieved therapy suspension.

Comorbidities recorded at inclusion included diabetes mellitus, IgM monoclonal gammopathy of unknown significance (MGUS), arterial hypertension, renal disease, previous thrombosis, gastrointestinal disorders, and other autoimmune diseases. For this analysis, only comorbidities present at treatment initiation were considered.

### Ethics

All participants provided written informed consent. The study was approved by the Ethics Committee of IRCCS Humanitas Clinical Institute (D.M. 8/2/2013; 413/17) and by local committees at all participating centres. De-identified data are available from the corresponding author upon reasonable request.

### Procedures

The primary objective was to evaluate induction and maintenance treatment patterns for CIDP across Italian centres and to identify their clinical and structural determinants. We examined inter-centre heterogeneity in treatment selection, demographic and clinical predictors of induction therapy, and the influence of organisational factors such as guideline adherence, economic constraints, and institutional prescribing practices. Temporal trends in prescribing behaviour were also assessed. Secondary objectives included characterising IVIg, SCIg, corticosteroids, PE, and immunosuppressants dosing strategies and identifying factors associated with their use.

Initiation dates of each patient's induction treatment were grouped into five-year periods to facilitate temporal comparisons and to broadly capture changes in clinical practice following major CIDP guideline publications (2006 EFNS/PNS, 2010 EFNS/PNS, 2021 EAN/PNS).^5^^,^^20^^,^^21^ Immunosuppressants use was categorised as IVIg-sparing or corticosteroids-sparing when introduced after a documented response to those agents, as rescue therapy when prescribed after failure of one or more first-line treatments, or as first-line therapy when initiated at diagnosis.^5^^,^^20^^,^^21^ Neonatal Fc receptor (FcRn) inhibitors were not approved for the treatment of CIDP in Italy during the study period and were therefore not included in the current analysis.

To complement registry data, a structured questionnaire was administered to all participating centres to further explore determinants of therapeutic decision-making and sources of inter-centre variability (Supplementary Table S1). The survey assessed the perceived influence of international guidelines, economic and organisational constraints, drug availability, clinician experience, patient-related clinical factors, and patient preferences on treatment selection. Additional items explored centre-level prescribing practices, reasons for switching treatments, and barriers to guideline implementation. Centre-level practices were operationally defined as the shared institutional clinical culture within each centre, encompassing local expert consensus, departmental traditions, and the training background of the medical staff (Supplementary Table S1). The questionnaire used neutral language, allowed multiple responses, and included ordered levels of influence (0 = no influence, 3 = major influence). One response per centre was collected and analysed descriptively to contextualise registry findings.

### Statistics

Categorical variables were reported as frequencies and percentages; continuous variables as means with SDs or medians with IQRs depending on distribution. Normality was tested with the Shapiro–Wilk test and inspection of histograms and Q–Q plots.

Group comparisons used chi-square or Fisher's exact tests for categorical variables. Continuous variables were analysed with independent-samples t-tests or one-way ANOVA (with Levene's test for homogeneity of variance) for normally distributed data, and with Wilcoxon–Mann–Whitney U or Kruskal–Wallis tests otherwise. Paired t-tests or Wilcoxon signed-rank tests were applied for within-patient comparisons. Associations between continuous variables were assessed using Pearson or Spearman correlation coefficients, as appropriate and reported as correlation coefficients (r) with p-values. Multinomial logistic regression was used with induction therapy as the dependent variable, using IVIg as reference treatment. To account for the clustering of patients within centres, a multilevel approach was adopted by incorporating centres as a random effect. Missing data were handled using a complete-case analysis approach.

Statistical significance was defined as a two-sided p-value <0.05. Univariate analyses were not adjusted for multiple comparisons, as their purpose was variable screening for multivariable modelling. Analyses were performed using IBM SPSS Statistics version 30.0 (IBM Corp., Armonk, NY, USA). Multinomial multilevel logistic regression was conducted using SAS version 9.4 M9 (SAS Institute Inc., Cary, NC, USA).

### Role of the Funding source

The funders had no role in study design, data collection, data analysis, data interpretation, or writing of the report.

## Results

**Supplementary Figure S2:**
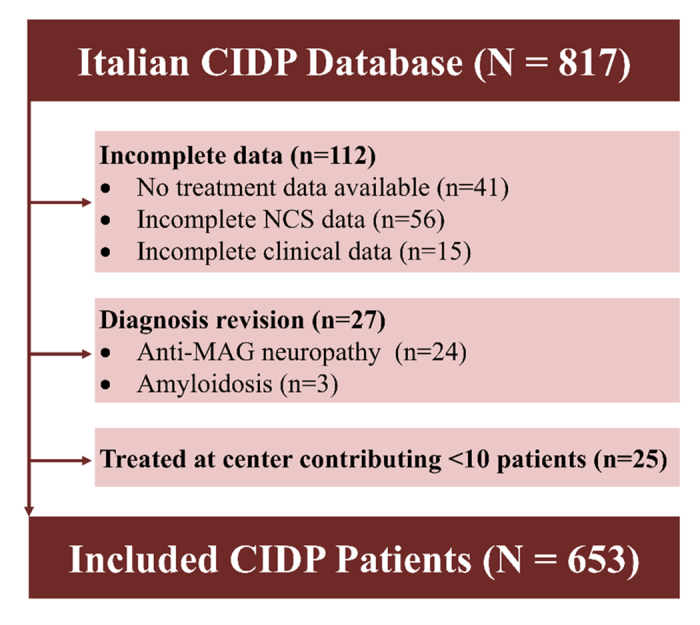
**Flowchart of population selection**.

Data collected up to July 2025 were included. As shown in Supplementary Figure S2, the initial cohort comprised 817 patients. A total of 112 were excluded due to incomplete data (*n* = 41), incomplete nerve conduction studies (*n* = 56), or missing clinical data (*n* = 15). An additional 27 were excluded following diagnostic revision (3 amyloidosis, 24 anti-myelin-associated-glycoprotein neuropathy). Twenty-five patients treated at centres contributing fewer than 10 cases were excluded because such small samples did not allow meaningful assessment of prescribing patterns. The final study population consisted of 653 patients from 24 tertiary referral centres (Supplementary Figure S2). Included and excluded patients were broadly similar in terms of key clinical features, except for a lower age at onset and shorter treatment delay among excluded patients (49.6 vs 55.4 years, U = 47.700, p = 0.00049, and 22.06 vs 38.37 months, U = 45.680, p = 0.010; Supplementary Table S2). A total of 11 patients (1.7%) tested positive for anti-(para)nodal IgG antibodies (7 anti-neurofascin-155 and 4 anti-contactin-1). The median diagnostic delay, defined as the time from symptom onset to diagnosis, was 9.4 months (IQR, 3.3–37.2). Diagnostic delay did not differ significantly across study periods (p = 0.371, Supplementary Table S3).

**Fig. 1:**
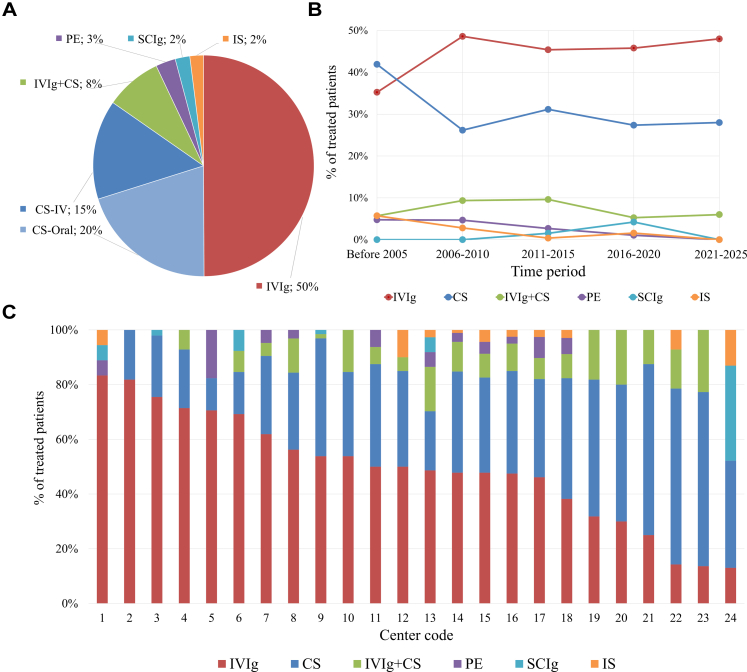
**Induction therapies in CIDP: overall distribution, temporal evolution, and inter-centre variability**. (A) Proportion of patients receiving each induction therapy among 653 cases. (B) Temporal trends in the selection of induction therapies. (C) Inter-centre heterogeneity in induction therapy choice across 24 Italian CIDP centres. Legend: CS, corticosteroid; IS, immunosuppressants; IVIg, intravenous immunoglobulin; PE, plasma exchange; SCIg, subcutaneous immunoglobulin.

We next examined induction treatment practices across the registry population, focussing on the determinants of therapeutic choice and their evolution over time. As illustrated in Fig. 1A, IVIg was the most frequently used induction therapy (326/653, 49.9%), followed by corticosteroids monotherapy (227/653, 34.8%), administered orally (132/653, 20.2%) or intravenously (95/653, 14.6%). Less frequently, patients received PE (19/653, 2.9%) or combined IVIg and corticosteroids (IVIg + CS, 54/653, 8.3%), including IVIg plus oral corticosteroids (46/653, 7.0%) and IVIg plus intravenous corticosteroids (8/653, 1.2%). A small proportion of patients received SCIg (14/653, 2.0%) or immunosuppressants (13/653, 2.0%) as induction monotherapy. Marked inter-centre heterogeneity in the selection of induction therapy was observed (p < 0.0001, Fig. 1C). IVIg use ranged from 13.0% to 83.3% across centres, while corticosteroids use varied from 0% to 64.3%. The use of PE and IVIg + CS combinations also differed substantially (0–17.7% and 0–22.7%, respectively). Temporal analysis (Fig. 1B) revealed significant shifts in prescribing patterns over time (χ^2^ = 38.559, p = 0.023). This trend remained significant after adjusting for individual centre participation (χ^2^ = 42.760, p = 0.0022), confirming a consistent evolution in practice across the network. IVIg use increased from 37.4% before 2005 (37/99) to 54.6% in 2021–2025 (24/44), whereas corticosteroids use declined from 45.5% (45/99) to 38.4% (17/44). SCIg use peaked at 5.2% in 2016–2020 (9/174) but decreased to 2.3% in 2021–2025 (2/44). PE and immunosuppressants use progressively declined and reached 0% in the most recent period. IVIg + CS combination therapy remained relatively stable over time. To identify which patient characteristics were associated with the choice of induction therapy, we first performed a univariate analysis (Table 1). IVIg was prescribed less frequently in patients with previous thrombosis (χ^2^ = 10.223, p = 0.046). Corticosteroids were used less often in pure motor CIDP (χ^2^ = 4.154, p = 0.035), A-CIDP (χ^2^ = 19.687, p = 0.0011), and diabetes mellitus (χ^2^ = 22.716, p = 0.00050). PE was more common in patients with IgM monoclonal gammopathy (χ^2^ = 17.264, p = 0.012) and in those with greater disability, reflected by higher INCAT scores (F = 8.337, p < 0.0001) and lower MRC sum scores (F = 8.337; p < 0.0001). To determine independent predictors, a multinomial logistic regression model (Model 1, Supplementary Tables S4 and S5) was constructed using IVIg as the reference category, including variables significant on univariate analysis with the referring centre as random effect. Independent clinical predictors included A-CIDP (F = 3.23, p = 0.0071), diabetes mellitus (F = 3.41, p = 0.0048), IgM MGUS (F = 2.32, p = 0.042). Other factors, including pure motor CIDP, MRC sum score, INCAT score, previous thrombosis, and gender, were not significant after adjustment.

**Table 1:** Clinical determinants of the selection of induction therapy (univariate analysis).

	Overall (n = 653)	IVIg (n = 326)	CS (n = 227)	IVIg and CS (n = 54)	PE (n = 19)	SCIg (n = 14)	IS (n = 13)	Statistics
Demographic								
Age at onset, mean (SD), years	49.6 (17.3)	50.0 (17.1)	49.1 (17.6)	50.0 (16.8)	44.4 (18.6)	52.2 (20.6)	50.2 (20.6)	F = 0.778 p = 0.779
Female gender, n (%)	229 (35.1%)	118 (36.2%)	69 (30.4%)	25 (46.3%)	10 (52.6%)	3 (21.4%)	4 (30.8%)	χ^2^ = 9.171 p = 0.102
CIDP Features, n (%)								
Acute onset	72 (11.1%)	39 (12%)	16 (7%)	7 (13%)	8 (42.1%)	2 (14.3%)	0 (0%)	χ^2^ = 19.687 p = 0.0011
Fulfilment of EFNS/PNS 2010 criteria	570 (87.3%)	285 (87.4%)	197 (86.8%)	50 (92.6%)	18 (94.7%)	10/(71.4%)	10 (76.9%)	χ^2^ = 6.479 p = 0.226
Typical CIDP	499 (76.4%)	245 (49%)	177 (35.5)	37 (7.4%)	18 (3.6%)	12 (2.4%)	10 (2.0%)	χ^2^ = 6.636 p = 0.238
Distal CIDP	47 (7.2%)	20 (42.6%)	20 (42.6%)	3 (6.4%)	0 (0%)	1 (2.1%)	3 (6.4%)	χ^2^ = 1.283 p = 0.263
Multifocal CIDP	37 (5.7%)	19 (51.4%)	14 (37.8%)	4 (10.8%)	0 (0%)	0 (0%)	0 (0%)	χ^2^ = 1.228 p = 0.275
Pure motor CIDP	32 (4.9%)	23 (71.9%)	5 (15.6%)	4 (36.4%)	0 (0%)	0 (0.0%)	0 (0%)	χ^2^ = 4.154 p = 0.039
Pure sensory cidp	27 (4.1%)	15 (55.6%)	9 (33.3%)	2 (7.4%)	0 (0%)	1 (3.7%)	0 (0%)	χ^2^ = 0.588 p = 0.479
Autoimmune (para)nodopathy	11 (1.7%)	4 (36.4%)	2 (18.2%)	4 (36.4%)	1 (9.1%)	0 (0%)	0 (0%)	χ^2^ = 1.507 p = 0.251
Comorbidities, n (%)								
IgM monoclonal gammopathy	30 (4.6%)	7 (2.1%)	16 (7%)	3 (5.6%)	3 (15.8%)	0 (0%)	1 (7.7%)	χ^2^ = 17.264 p = 0.012
Previous thrombosis	14 (2.1%)	3 (0.9%)	9 (4%)	0 (0%)	1 (5.3%)	0 (0%)	1 (7.7%)	χ^2^ = 10.223 p = 0.046
Kidney disease	8 (1.2%)	3 (0.9%)	4 (1.8%)	1 (1.9%)	0 (0%)	0 (0%)	0 (0%)	χ^2^ = 2.337 p = 0.690
Paediatric onset	31 (4.7%)	14 (4.3%)	14 (6.2%)	1 (1.9%)	1 (5.3%)	0 (0%)	1 (7.7%)	χ^2^ = 2.871 p = 0.611
Diabetes mellitus	70 (10.7%)	48 (14.7%)	11 (4.8%)	3 (5.6%)	1 (5.3%)	3 (21.4%)	4 (30.8%)	χ^2^ = 22.716 p = 0.00050
Arterial hypertension	175 (26.8%)	98 (30.1%)	56 (24.7%)	15 (27.8%)	3 (15.8%)	1 (7.1%)	2 (15.4%)	χ^2^ = 6.579 p = 0.256
Gastritis	15 (2.3%)	10 (3.1%)	4 (1.8%)	0 (0%)	0 (0%)	1 (7.1%)	0 (0%)	χ^2^ = 3.733 p = 0.476
Disease severity, mean (SD)								
MRC sum score, mean	53.1 (7.1)	53.2 (8.1)	55.0 (5.3)	51.0 (6.0)	45.3 (10.5)	53.0 (5.6)	52.0 (4.7)	F = 3.154 p = 0.0087
INCAT, mean	2.6 (1.9)	2.5 (1.9)	2.0 (1.9)	4.0 (2.2)	4.9 (2.4)	2.8 (1.7)	3.0 (1.5)	F = 8.337 p < 0.0001

LEGEND: CIDP, chronic inflammatory demyelinating polyradiculoneuropathy; CS, corticosteroids; European Federation of Neurological Societies/Peripheral Nerve Society, EFNS/PNS; INCAT, inflammatory neuropathy cause and treatment score; IVIg, intravenous immunoglobulins; MRC sum score, medical research council sum score; PE, plasma exchange.

**Fig. 2:**
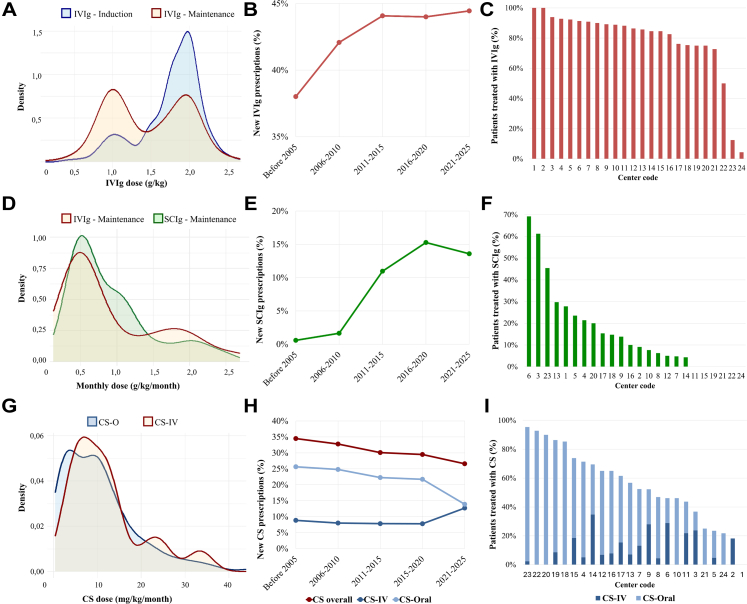
**Use of IVIg, SCIg and corticosteroid in CIDP**. (A) Distribution of IVIg dosage (g/kg) in induction vs maintenance treatment. (B) Temporal trend of overall IVIg prescriptions. (C) Proportion of patients receiving IVIg at any point during their disease course, by treating centre. (D) Distribution of IVIg and SCIg maintenance dosage (g/kg/month). (E) Temporal trend of overall SCIg prescriptions. (F) Proportion of patients receiving SCIg at any point during their disease course, by treating centre. (G) Distribution of oral and intravenous CS dosages (mg/kg) at meintenance. (H) Temporal trends of overall CS, oral CS, and intravenous CS prescriptions. (I) Proportion of patients receiving CS at any point during their disease course, by treating centre. LEGEND: CS, corticosteroid; CS-IV, intravenous corticosteroids; CS-O, oral corticosteroids; IVIg, intravenous immunoglobulins; SCIg, subcutaneous immunoglobulins.

**Supplementary Figure S3:**
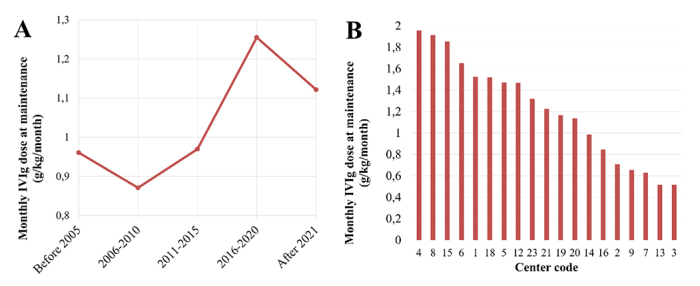
**IVIg dosage at maintenance: trend in time and mean dosage among centers**.

Among patients receiving immunoglobulin therapy, both intravenous and subcutaneous formulations showed distinct prescribing trajectories over the study period. A total of 522 patients (79.9%) received IVIg during their disease course. Its overall use increased modestly over time, from 38.0% before 2005 (65/171) to 44.3% in 2021–2025 (35/79) (Fig. 2B), with substantial inter-centre variability (Fig. 2C). The median induction dose was 1.88 g/kg (IQR 1.6–2.0), with a bimodal distribution characterised by a predominant peak at 2 g/kg and a smaller secondary peak at 1 g/kg (Fig. 2A). At maintenance, the median dose decreased to 1.33 g/kg (IQR 0.98–1.95; t = 10.826, p < 0.0001), but retained a similar bimodal pattern, reflecting heterogeneous dosing practices across centres. The median inter-infusion interval was 35 days (IQR 30–60), corresponding to a median monthly dose of 0.80 g/kg (IQR 0.5–1.47). No clinical or demographic predictors of maintenance IVIg dosing were identified (Supplementary Table S6). However, treatment initiation in more recent time periods was associated with higher monthly doses (R = 0.220, p < 0.001; Supplementary Figure S3A), and inter-centre differences remained pronounced (Z = −9.133, p < 0.0001; Supplementary Figure S3B). Among patients initially treated with IVIg, 107 (20.5%) were switched to SCIg; of these, 19 (17.8%) subsequently worsened or returned to IVIg. Switching occurred after a median of 23.4 months (IQR 10.2–80.2), with marked centre-level variation: some centres switched after a median of 46.9 months (IQR 10.1–96.9), whereas others transitioned patients as early as 6.7 months (IQR 3.1–54.4). Switching the route of administration did not significantly affect overall monthly immunoglobulin exposure, which remained similar before and after switching [0.53 g/kg/month (IQR 0.47–1.52) under IVIg vs 0.70 g/kg/month (IQR 0.50–1.14) under SCIg; Z = −0.159, p = 0.874; Fig. 2D]. SCIg use increased steadily until 2020, peaking at 15.2% of new prescriptions (42/277), with a slight decline thereafter (Fig. 2E). Although its overall adoption remained limited, inter-centre variability was considerable: some centres used SCIg in up to 75% of IVIg responders, whereas others did not use it at all (Fig. 2F).

Turning to corticosteroid use, both the frequency and the route of administration evolved substantially across the study period. A total of 376 patients (57.6%) received corticosteroids therapy during their disease course. Among them, oral corticosteroids were prescribed in 250 (66.5%) patients, intravenous corticosteroids followed by an oral tapering in 62 (16.5%), and exclusively intravenous corticosteroids in 64 (17.0%). Overall corticosteroids use showed a modest temporal decline, accompanied by a shift in prescribing patterns: oral corticosteroids decreased from 25.7% before 2005 (44/171) to 13.9% (11/79) in 2021–2025, while intravenous corticosteroids increased from 8.8% (15/171) to 12.7% (10/79) over the same period (Fig. 2H). Substantial inter-centre heterogeneity was observed in both overall corticosteroids use and preferred administration route (Fig. 2I). Some centres relied predominantly on oral corticosteroids, whereas others almost exclusively used intravenous corticosteroids, reflecting the absence of a standardised approach. The median oral corticosteroids induction dose was 0.68 mg/kg/day (IQR 0.51–0.86), decreasing to 0.30 mg/kg/day (IQR 0.13–0.45) for maintenance. For intravenous corticosteroids, the median induction dose per cycle was 2000 mg (IQR 1000–3000), decreasing to 917 mg (IQR 500–1125) at maintenance, with a median inter-administration interval of 30 days (IQR 30–120; Fig. 2G). Distribution curves indicated overall consistency in cumulative corticosteroids exposure across oral and intravenous regimens when adjusted for patient weight and time.

**Fig. 3:**
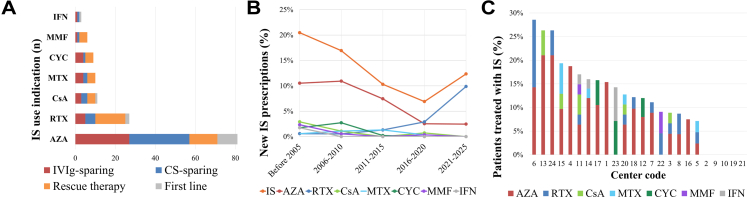
**Use of immunosuppressants**. (A) Number of patients treated with each immunosuppressant, stratified by indication (first-line, rescue therapy, or as IVIg/CS-sparing agents). (B) Temporal trends in new IS prescriptions and distribution of individual drugs over time. (C) Inter-centre variability in IS use, expressed as the proportion of patients treated with each immunosuppressive drug at each centre (overall use during the disease course). LEGEND: AZA, azathioprine; CS, corticosteroids; CsA, cyclosporine; CYC, cyclophosphamide; IFN, interferon; IS, immunosuppressors; IVIg, intravenous immunoglobulin; MMF, mycophenolate mofetil; MRX, methotrexate; RTX, rituximab.

Beyond immunoglobulins and corticosteroids, the use of immunosuppressant agents and plasma exchange showed notable temporal changes. Immunosuppressants were prescribed in 121 patients (18.5%) during the disease course. Azathioprine was the most frequently used agent (81/653, 12.4%), typically as an IVIg- or corticosteroids-sparing regimen (27 and 30 patients, respectively). Azathioprine used as a sparing regimen resulted in successful suspension of first-line therapy in 24 instances (24/57, 42.1%), while first-line regimens were maintained in 14 cases (14/57, 24.6%) and disease deterioration or resumption of first-line therapy occurred in 19 cases (19/57, 33.3%). Adverse events occurred in 15 patients (15/81, 18.5% of cases). Rituximab was prescribed in 27 patients (4.1%), mainly as rescue therapy following failure of first-line treatments (*n* = 15). When used as rescue therapy, improvement was observed in 10 patients (10/15, 66.6%). Two patients with autoimmune (para)nodopathy (one anti-contactin-1 and one anti-neurofascin-155) were treated with rituximab, achieving disease control and discontinuation of other immunomodulatory therapies. Rituximab use was associated with adverse events in 9 patients (9/27, 33.3%). Rituximab induction followed two main dosing protocols: two 1000 mg infusions 2 weeks apart (*n = 23*) or 375 mg/m^2^ weekly for 4 consecutive weeks (*n = 4*). Maintenance regimens varied, with most patients re-treated at 6 months (n = 17), a few on individualised schedules (n = 3), and 7 receiving no maintenance (5 due to lack of induction efficacy). Cyclosporine was used in 11 patients (1.7%), whereas methotrexate, cyclophosphamide, mycophenolate mofetil, and interferon were rarely prescribed (Fig. 3A). Overall response and adverse events rates to immunosuppressants are summarised in Supplementary Table S7. Overall immunosuppressants use declined over time, except for rituximab. Rituximab prescriptions increased progressively, reaching levels comparable to azathioprine by 2016–2020 (8/277, 2.9% of new therapies recorded in the registry) and becoming the most commonly used immunosuppressant in 2021–2025 (8/79, 10.1% of new prescriptions; Fig. 3B). Marked inter-centre heterogeneity was observed in both the frequency of immunosuppressants use and the preferred agent, with some centres relying primarily on azathioprine, whereas others favoured rituximab or cyclosporine (Fig. 3C). PE was used in 62 patients (9.5%), mainly in earlier decades, and declined from 6.4% (11/171) before 2005 to 1.3% (1/79) after 2021. The most common indication was lack of response to previous therapies (30/62, 48.4%), followed by induction treatment (19/62, 30.6%), partial response (9/62, 14.5%), and relapse treatment (4/62, 6.5%).

We then compared treatment outcomes across induction strategies with respect to therapy response, suspension, and residual disability. Table 2 summarises treatment outcomes across induction strategies, treatment modalities, CIDP phenotypes, and study periods. Across treatment modalities, induction therapy elicited a therapeutic response in 75.3% of patients (492/653), with the highest rates observed for IVIg (281/326, 86.2%) and the lowest for immunosuppressants and SCIg [38.5% (5/13) and 57.1% (8/14), respectively; χ^2^ = 60.001; p < 0.0001, Table 2]. PE and corticosteroids were associated with lower response rates [57.9% (11/19) and 62.1% (141/227), respectively], whereas combined IVIg + CS achieved response rates comparable to IVIg alone (46/54, 85.2%). These patterns were also reflected in the number of treatment attempts required to achieve response, which was lowest with IVIg [mean 1.17 (SD 0.55)] and highest with corticosteroids and PE [1.49 (0.77) and 1.79 (1.32), respectively; F = 5.795; p < 0.0001, Table 2]. The number of attempts was similarly low for SCIg and immunosuppressants. Induction strategies associated with lower response rates were also associated with less favourable long-term outcomes. Use of a reduced IVIg induction dose (<2 g/kg) was associated with lower rates of achievement of minimal residual disability at last follow-up [29.9% (49/164) vs 40.9% (65/162); χ^2^ = 4.120; p = 0.042, Table 2] and therapy suspension [41.5% (68/164) vs 54.9% (89/162); χ^2^ = 5.945; p = 0.015, Table 2]. Oral corticosteroids were associated with significantly lower rates of therapy suspension than intravenous corticosteroids [25.8% (34/132) vs 54.7% (52/95); χ^2^ = 18.502; p < 0.0001, Table 2). Long-term outcomes also differed across CIDP variants. Patients with typical CIDP suspended therapy in 41.9% of cases (208/499), whereas those with motor CIDP were least likely to do so (7/32, 21.9%, χ^2^ = 26.729; p < 0.0001, Table 2) and those with distal CIDP most likely (34/47, 72.3%). Achievement of minimal residual disability at last follow-up was highest in distal CIDP (30/47, 63.8%) and lowest in autoimmune (para)nodopathies (1/11, 9.1%). In these patients, induction therapy had been initiated before antibody testing was performed, and therefore treatment selection was not influenced by antibody status. Among the small subgroup of patients with motor CIDP who received corticosteroids as induction therapy (n = 5), long-term outcomes appeared broadly similar to those of patients initially treated with IVIg (Table 2), although interpretation is limited by sample size. Over time, clinical outcomes improved across the study period. Induction response rates increased from 67.7% (67/99) before 2005 to 88.6% (39/44) after 2021 (p = 0.016, Table 2), accompanied by higher rates of therapy suspension [39.4% (39/99) to 45.5% (29/44), Z = −2.457; p = 0.014] and minimal residual disability at last follow-up [31.3% (31/99) to 51.2% (22/44), Z = −2.149; p = 0.034], together with a reduction in the number of treatment attempts required to achieve response [mean 1.50 (SD 0.99) before 2005 vs 1.20 (1.00) after 2021; β = −0.135, p = 0.0016].

**Table 2:** Outcome comparison across treatment modalities.

	Therapeutic delay, months (SD)	Treatment outcomes			
		Induction response, n/treated (%)	Treatment attempts before response, mean (SD)	Suspended therapy, n/treated (%)	Minimal residual disability, n/treated (%)
Whole population (n = 653)	38.37 (69.77)	492 (75.3%)	1.30 (0.71)	288 (44.1%)	235 (36.3%)
Induction treatment					
IVIg (n = 326)	40.26 (78.39)	281 (86.2%)^a^	1.17 (0.55)^a^	157 (48.2%)	114 (35.4%)
CS (n = 227)	40.39 (64.29)	141 (62.1%)^a^	1.49 (0.77)^a^	93 (41.0%)	90 (39.8%)
IVIg + CS (n = 54)	27.97 (53.46)	46 (85.2%)^a^	1.29 (0.87)^a^	16 (29.6%)	18 (33.3%)
PE (n = 19)	14.50 (28.11)	11 (57.9%)^a^	1.79 (1.32)^a^	10 (52.6%)	4 (21.1%)
SCIg (n = 14)	46.52 (48.52)	8 (57.1%)^a^	1.00 (0.00)^a^	5 (35.7%)	6 (42.9%)
IS (n = 13)	16.65 (23.25)	5 (38.5%)^a^	1.23 (0.44)^a^	7 (53.8%)	3 (21.1%)
Treatment modalities					
Reduced IVIg dose (<2 g/kg)					
Yes (n = 164)	43.3 (82.1)	144 (87.8%)	1.14 (0.5)	68 (41.5%)^b^	49 (29.9%)^b^
No (n = 162)	37.2 (74.5)	137 (84.6%)	1.20 (0.6)	89 (54.9%)^b^	65 (40.9%)^b^
Motor CIDP treated with CS at induction					
Yes (n = 5)	13.29 (11.80)	4 (80%)	1.25 (0.50)	1 (20.0%)	1 (20%)
No (n = 28)	29.47 (58.82)	22 (81.5%)	1.48 (1.19)	6 (22.2%)	9 (33.3%)
CS route at induction					
Oral (n = 132)	36.15 (62.52)	77 (58.3%)	1.53 (0.72)	34 (25.8%)^c^	51 (38.6%)
IV (n = 95)	47.20 (72.02)	57 (60.0%)	1.48 (0.92)	52 (54.7%)^c^	35 (36.8%)
Clinical presentation					
Typical (n = 499)	38.86 (71.82)	376 (75.4%)	1.31 (0.68)	208 (41.9%)^d^	164 (33.1%)^d^
Distal CIDP (n = 47)	48.72 (78.50)	41 (87.2%)	1.23 (0.86)	34 (72.3%)^d^	30 (63.8%)^d^
Multifocal CIDP (n = 37)	36.62 (52.64)	29 (78.4%)	1.31 (0.66)	16 (43.2%)^d^	15 (42.9%)^d^
Pure-motor (n = 32)	27.32 (55.03)	26 (78.4%)	1.45 (1.12)	7 (21.9%)^d^	10 (31.3%)^d^
Pure-sensory (n = 27)	41.44 (65.62)	21 (77.8%)	1.14 (0.36)	17 (63%)^d^	15 (55.6%)^d^
Autoimmune (para)nodopathy (n = 11)	5.88 (6.08)	8 (72.7%)	1.27 (0.47)	5 (45.5%)^d^	1 (9.1%)^d^
Timeframe					
Before 2005 (n = 99)	39.91 (62.55)	67 (67.7%)^e^	1.50 (0.99)^e^	39 (39.4%)^e^	31 (31.3%)^e^
2006–2010 (n = 98)	32.47 (51.34)	68 (69.4%)^e^	1.32 (0.55)^e^	38 (38.8%)^e^	28 (28.6%)^e^
2011–2015 (n = 238)	40.02 (71.94)	183 (76.9%)^e^	1.31 (0.62)^e^	98 (41.2%)^e^	92 (38.7%)^e^
2016–2020 (n = 174)	41.23 (83.38)	144 (82.8%)^e^	1.18 (0.64)^e^	93 (53.4%)^e^	62 (36.5%)^e^
2021–2025 (n = 44)	28.78 (47.67)	39 (88.6%)^e^	1.20 (1.00)^e^	20 (45.5%)^e^	22 (51.2%)^e^
Statistics		a(χ^2^ = 60.001, p < 0.0001) e(Z = −3.780, p = 0.0016)	a(F = 5.795, p < 0.0001) e(β = −0.135, p = 0.0012)	b(χ^2^ = 5.945, p = 0.015) c(χ^2^ = 18.502, p < 0.0001) d(χ^2^ = 26.729, p < 0.0001) e(Z = −2.457, p = 0.014)	b(χ^2^ = 4.120, p = 0.042) d(χ^2^ = 26.514, p < 0.001); e(Z = −2.149, p = 0.034)

LEGEND: IVIg, intravenous immunoglobulins; CS, corticosteroids; PE, plasma exchange; SCIg, Subcutaneous immunoglobulins; IS, immunosuppressors; INCAT, inflammatory neuropathy cause and treatment score; RODS, Rasch-built Overall Disability Scale; MRCss, medical research council sum score.

a Comparison of outcomes by induction treatment groups.

b Comparison of outcomes by reduced IVIg dose (<2 g/kg, Yes vs No).

c Comparison of outcomes by corticosteroid route at induction (oral vs intravenous).

d Comparison of outcomes across clinical presentation subtypes.

e Comparison of outcomes across time periods.

**Fig. 4:**
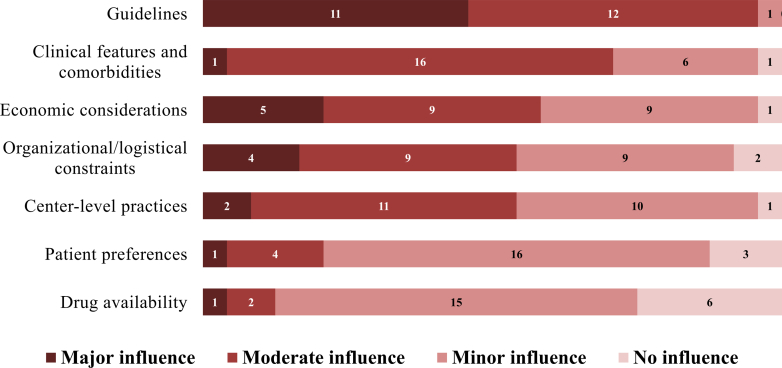
**Questionnaire results of key aspects of therapeutic decision-making**. Values indicate the number of centres reporting each response category. The survey explored several domains relevant to treatment selection in CIDP. Respondents were asked: to what extent international guidelines influence therapeutic choices; how influential patient-specific clinical features and comorbidities are in guiding decisions (subcategory: overall); to what degree economic considerations, such as drug price, reimbursement policies, and overall economic sustainability, affect treatment choices; and to what extent organisational or logistical constraints (e.g., access to day-care infusion facilities, nursing resources, administrative hurdles) impact clinical decision-making. Additional questions assessed whether treatment choices are shaped by a shared “centre approach,” including local consensus, traditions, or training background, rather than solely by individual clinicians; how much patient preferences (e.g., home vs hospital therapy, oral vs infusion, lifestyle factors) influence therapy selection; and how often decisions are limited by the local availability of specific therapies (subcategory: overall). For the exact survey items, see Supplementary Table S1.

**Supplementary Figure S4:**
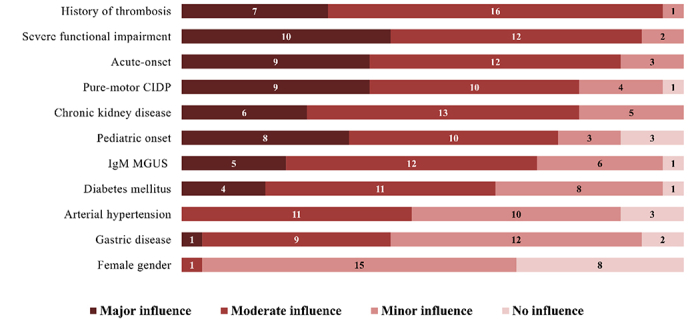
**Questionnaire response—Influence of clinical factors and comorbidities**.

Finally, to contextualise the observed prescribing patterns, we examined the results of a structured survey administered to participating centre coordinators regarding the factors influencing local treatment decisions. Survey responses indicated that international clinical guidelines were the most influential determinant of treatment decisions (moderate-to-major influence in 23/24 centres, 95.8%), followed by patient clinical features and comorbidities (17/24, 70.8%) (Fig. 4). Structural factors also played a substantial role: economic considerations influenced prescribing in 14/24 (58.3%), and organisational/logistical constraints in 13/24 centres (54.2%) (Fig. 4). Centre-level practices were also influential, being rated as moderate-to-major influence in 13/24 centres (54.2%), whereas patient preferences and drug availability were generally considered minor contributors [moderate-to-major influence in 5/24 (20.8%) and 3/24 (12.5%) centres, respectively] (Fig. 4). Among clinical features, a history of thrombosis (23/24, 95.8%), severe disability (22/24, 91.7%), acute-onset presentation (21/24, 87.5%), pure motor CIDP, and chronic kidney disease (each 19/24, 79.2%; Supplementary Figure S4) were most commonly rated as major or moderate determinants of therapy selection. Regarding treatment constraints, IVIg availability was most often reported as limited (often/very often) in 11/24 centres (45.8%), followed by SCIg (10/24, 41.7%) and PE (8/24, 33.3%), whereas limitations for corticosteroids and immunosuppressants were rare (Supplementary Table S1). The main reasons for switching treatment were insufficient efficacy (19/24, 79.2%), followed by patient preference or convenience (17/24, 70.8%), and adverse events (14/24, 58.3%), while economic or healthcare-related drivers were less frequent (9/24, 37.5%; Supplementary Table S1). Administrative or organisational barriers were the most commonly cited obstacle to guideline implementation (15/24, 62.5%), whereas unclear or insufficient evidence in guidelines was reported by only 5/24 centres (20.8%) (Supplementary Table S1). Furthermore, in an exploratory analysis, we integrated in a second multinomial model the level of influence from questionnaire-derived centre characteristics. This approach allowed identification of the structural or cultural drivers of centre variability (Model 2, Supplementary Table S4). Organisational/logistical constraints (F = 2.55, p = 0.027), and patient-specific clinical features (F = 3.39, p = 0.0051) were independently associated with induction therapy choice. Clinical predictors from Model 1 remained significant, except IgM monoclonal gammopathy without anti-MAG antibodies.

## Discussion

This multicentre study provides real-world evidence on CIDP treatment patterns in Italy, highlighting three major findings: marked inter-centre heterogeneity in therapeutic choices, a progressive evolution of prescribing behaviour over two decades, and the presence of a minority of patients receiving treatments that deviate from guideline recommendations.

Substantial variability was observed across nearly all aspects of CIDP management, including the choice of induction therapy, IVIg loading and maintenance dosing, SCIg adoption, corticosteroids use and route of administration, and the overall employment of immunosuppressants. Variability extended to specific immunosuppressant agents, their clinical indications, and rituximab dosing protocols. These findings are consistent with previous reports, mostly from surveys or limited cohorts, which have similarly documented wide differences in CIDP treatment practices across countries and healthcare systems.^15^, ^16^, ^17^, ^18^, ^19^ Such heterogeneity may have several implications. Scientifically, it underscores the absence of validated biomarkers or stratification tools capable of guiding individualised therapy and highlights the need for comparative-effectiveness studies to clarify which patients benefit most from specific regimens. Clinically, variability may result in undertreatment, overtreatment, inappropriate escalation, or delays in optimisation, particularly in relation to IVIg dosing, corticosteroids schedules, and the use of IS. In our cohort, this variability was also reflected in long-term outcomes, as lower remission rates and reduced likelihood of treatment suspension were observed in patients receiving a reduced IVIg induction dose (<2 g/kg) and in those treated with oral corticosteroids. However, these associations should be interpreted cautiously, as treatment allocation in this observational cohort was not randomised and may have been influenced by clinical phenotype, disease severity, and physician preference. At a health-system level, inconsistent prescribing may contribute to inefficient resource use, increased costs, and unequal access to optimal therapies, especially given the economic burden of long-term immunoglobulin treatment.^4^

The survey findings shed light on the determinants of this heterogeneity. Although clinicians consistently endorsed international guidelines and patient clinical features as primary drivers of treatment decisions, they also acknowledged constraints related to economic considerations, drug availability, organisational barriers, and local prescribing culture. The weight of centre-level practices reported in the survey (encompassing local tradition and training) highlights the role of institutional practice patterns in treatment selection. These insights align with the multivariable models, which showed that contextual factors, rather than clinical profile alone, exerted an independent influence on therapeutic allocation. The observed inter-centre heterogeneity should also be interpreted in the context of Italy's decentralised healthcare system, where 21 regional entities implement distinct reimbursement models for high-cost drugs. In regions using dedicated pharmaceutical funds (often referred to as ‘File F’), IVIg costs are decoupled from hospital budget. Conversely, in regions where costs must be absorbed within fixed Diagnosis Related Group (DRG) payments for inpatient or day-hospital stays, the high acquisition price of IVIg frequently exceeds the reimbursement tariff. These structural financial differences may contribute to inter-centre variability in IVIg prescribing. Together, these observations indicate that variability in CIDP management arises not only from differences in disease presentation but also from structural disparities across centres and health-system environments.

A relevant finding was the use of therapies outside current guideline recommendations. The most frequent deviation involved the combined use of IVIg and corticosteroids as induction therapy. Although this approach may be intended to enhance therapeutic efficacy, recent evidence from the OPTIC randomised controlled trial showed that adding intravenous methylprednisolone to IVIg did not significantly increase remission rates compared with IVIg alone and that the study was prematurely terminated because of thromboembolic events in the combination group. While some secondary outcomes suggested greater improvement with combined therapy, the overall benefit–risk balance of this strategy remains uncertain.^26^ Additional deviations included the use of SCIg or immunosuppressants as induction therapies, for which evidence is limited and regulatory approval lacking.^5^^,^^20^^,^^21^ These practices may increase the risk of undertreatment or delayed improvement. Interestingly, although SCIg and immunosuppressants showed lower response rates when used as induction therapies, they were associated with relatively few treatment attempts before response. This may reflect a tendency to maintain these therapies longer before declaring failure, particularly for immunosuppressants whose effects may take months to emerge. The small subgroup size and selective clinical use (e.g., contraindications to IVIg or corticosteroids) may also have contributed to this pattern. Another deviation involved using an IVIg loading dose of 1 g/kg instead of the guideline-recommended 2 g/kg.^5^^,^^20^^,^^21^ Given the established efficacy of the standard dose, such reductions may compromise early disease control and delay optimal disease stabilisation.

This study also documents the temporal evolution of CIDP treatment practices. Over the 20-year period examined, use of IVIg as induction therapy increased significantly, alongside a gradual rise in maintenance dosing. This trend likely reflects growing confidence in IVIg following guideline updates, accumulating clinical experience, and possibly improved access.^5^, ^6^, ^7^^,^^19^^,^^20^ In parallel, SCIg use increased until 2020, likely influenced by the COVID-19 pandemic, which favoured home-based therapies, before stabilising. However, SCIg adoption remained limited and highly variable across centres, emphasising persistent implementation barriers in Italy, including administrative constraints, logistical challenges, and reimbursement limitations. The only moderate decline in corticosteroids use underscores their continued role as a cost-effective and easily administered alternative. Corticosteroids use evolved, with increased uptake of intravenous pulse regimens, consistent with evidence from trials such as PREDICT showing better short-term outcomes and tolerability compared with daily oral regimens.^8^ Nevertheless, intravenous corticosteroids remain underused, possibly reflecting a persistent preference for oral administration despite its slower onset and less favourable side–effect profile.^8^

Immunosuppressant use declined over time, particularly for traditional agents, while rituximab prescriptions increased steadily. This pattern likely reflects growing observational evidence supporting rituximab in selected or refractory CIDP cases.^27^, ^28^, ^29^, ^30^, ^31^ However, a recent randomised controlled trial failed to demonstrate efficacy in IVIg-responsive patients, though signals of benefit in selected outcomes remain.^32^ The virtual disappearance of PE as an induction choice, and its marked decline overall, contrasts with its established evidence from clinical trials. This trend is likely driven by significant logistical hurdles, including the need for specialised equipment and coordination with centralised aphaeresis units, as well as by the requirement for reliable vascular access. As highlighted in current EAN/PNS guidelines, these practical limitations contribute to positioning PE as a third-line option after IVIg and corticosteroids, despite its proven efficacy.^5^ These factors may therefore have contributed to the repositioning of PE as a rescue therapy for refractory cases or severe relapses rather than a primary option for induction or maintenance.^5^ Consistent with these shifts in practice, clinical outcomes in our study improved over time, with higher response rates and increased therapy suspension in more recent study periods.

Finally, while our five-year periods are anchored to international guidelines, they also capture broader shifts in the Italian healthcare landscape. These include, for example, the post-2010 reimbursement of SCIg, the logistical constraints on hospital-based care during the COVID-19 pandemic, and regional drug shortages or changes in regulatory policies that may not strictly align with our defined intervals.

These findings suggest several practical implications. Reducing unwarranted inter-centre heterogeneity will require not only active dissemination and local implementation of evidence-based guidelines, but also the identification and mitigation of non-clinical barriers (including logistical, administrative, and reimbursement-related constraints) that may limit treatment access. Treatment strategies outside current guideline recommendations should also be systematically monitored, as their use highlights areas where evidence remains limited and where future guidelines may need to provide more explicit guidance. The temporal evolution of prescribing practices further suggests that flexible models of care are needed to rapidly adapt to changes in treatment availability, prescribing patterns, and emerging therapeutic strategies. Such measures may help promote more standardised and equitable care while preserving individualised treatment decisions.

Several limitations merit consideration. First, unmeasured confounding may have influenced prescribing patterns. Second, the observational design precludes causal inference regarding the determinants of treatment selection or outcomes. Third, although survey data provided valuable insight into centre-level practices, responses reflect subjective perceptions and may be affected by reporting bias. Finally, organisational and reimbursement structures specific to Italy likely shaped treatment patterns; however, similar heterogeneity has been consistently reported in studies from other countries, indicating that this variability represents a broader international phenomenon rather than an Italian anomaly.^15^, ^16^, ^17^, ^18^, ^19^

In conclusion, this study offers a comprehensive assessment of CIDP treatment practices across multiple centres, revealing substantial heterogeneity, clear temporal evolution, and non-negligible deviations from guideline recommendations. These findings underscore the need for improved standardisation, better alignment with evidence-based recommendations, and health-system initiatives aimed at reducing unwarranted variability. As new and costly therapies emerge, ensuring equitable, efficient, and guideline-consistent care will be essential for optimising outcomes in CIDP.

## Contributors

ADL conceived and designed the study, coordinated data collection, performed the analyses, and draughted the manuscript. FM, DC, CB, YF, AM, AS, VDS, GC, GAM, LB, MC, MF, LL, MLu, SM, GP, TR, MLc, GS, GLP, GC, MI, TC, FN, DR, FH, EV contributed to study conception and design, data acquisition, and interpretation of results, and critically revised the manuscript for important intellectual content. EB performed part of the statistical analysis and critically revised the manuscript for important intellectual content. PED and ENO supervised the study, contributed to study design, reviewed the statistical analyses, and critically revised the final version of the manuscript. ADL, PED and ENO have verified the data. All authors approved the final manuscript and agreed to be accountable for all aspects of the work.

## Data sharing statement

Deidentified data used for this study are available upon reasonable request from the corresponding author. No custom analysis code or proprietary software was developed for this study. Statistical analyses were performed using IBM SPSS Statistics version 30.0 and SAS version 9.4 M9, both commercially available platforms. Analysis syntax can be made available upon reasonable request from the corresponding author.

## Declaration of interests

Alberto De Lorenzo received travel grants to attend scientific meetings from Kedrion. Pietro Emiliano Doneddu reports personal fees for Advisory from ArgenX, and received travel grants to attend scientific meetings from CSL Behring, and Kedrion. Giuseppe Cosentino has received travel grants to attend scientific meetings from CSL Behring, and Kedrion. Massimiliano Filosto has served on scientific advisory boards for CSL Behring, Sanofi, and Amicus, and has received travel grants from Sanofi, Biogen, Kedrion, and CSL Behring to attend scientific meeting. Anna Mazzeo has received travel grants from Kedrion, and CSL Behring to attend scientific meeting. Maurizio Inghilleri has received travel grants to attend scientific meetings from CSL Behring, ArgenX, and Alexion. Vincenzo Di Stefano received support for travels for attending meetings from Alexion, Alnylam, Argenx, UCB; received compensation for speaking from Alexion, UCB, Argenx and Alnylam; is Sub-Investigator in clinical trials for Alexion, Alnylam, Argenx, and Sanofi. Eduardo Nobile-Orazio reports personal fees for Advisory or Scientific Board from ArgenX—Belgium, Takeda–Italy and USA, CSL-Behring–Italy and USA, Janssen—USA, Kedrion—Italy, LFB—France, Roche—Switzerland, Sanofi–USA. The other authors declare no conflict of interest.
