# Supplementary material for: Inter-centre heterogeneity, temporal evolution, and factors associated with treatment selection and outcomes in chronic inflammatory demyelinating polyradiculoneuropathy: a multicentre, combined prospective and retrospective observational study
Source: eClinicalMedicine. 2026 Jun 23;97:104031. doi: 10.1016/j.eclinm.2026.104031 (PMC13316210; doi:10.1016/j.eclinm.2026.104031)
Supplement: Supplementary Table S1 [file mmc1.docx]

# **Table S1 – Questionnaire about prescription practices and guideline**

| **Question** | **Options and Results** |
| --- | --- |
| **1. Guideline adherence**  To what extent have international guidelines (EFNS/PNS 2006, 2010; EAN/PNS 2021) influenced treatment choices in CIDP in your center? | - Major influence: 11/24 (45·8%) - Moderate influence: 12/24 (50·0%) - Minor influence: 1/24 (4·2%) - No influence: 0/24 (0·0%) |
| **2. Economic considerations**  To what degree do treatment costs (drug price, reimbursement policies, overall economic sustainability) influence therapeutic choices in your center? | - Major influence: 5/24 (20·8%) - Moderate influence: 9/24 (37·5%) - Minor influence: 9/24 (37·5%) - No influence: 1/24 (4·2%) |
| **3.** **Drug availability**  How often are treatment decisions limited by the local availability of specific therapies in your center? |  |
| Overall | - Very often: 1/24 (4·2%) - Often: 2/24 (8·3%) - Sometimes: 15/24 (62·5%) - Rarely/never: 6/24 (25·0%) |
| IVIg | - Very often: 5/24 (20·8%) - Often: 6/24 (25·0%) - Sometimes: 9/24 (37·5%) - Rarely/never: 4/24 (16·7%) |
| SCIg | - Very often: 5/24 (20·8%) - Often: 5/24 (20·8%) - Sometimes: 6/24 (25·0%) - Rarely/never: 8/24 (33·3%) |
| Corticosteroids | - Very often: 1/24 (4·2%) - Often: 1/24 (4·2%) - Sometimes: 2/24 (8·3%) - Rarely/never: 20/24 (83·3%) |
| Plasma exhange | - Very often: 8/24 (33·3%) - Often: 0/24 (0·0%) - Sometimes: 6/4 (150·0%) - Rarely/never: 10/24 (41·7%) |
| Immunesuppressants | - Very often: 1/24 (4·2%) - Often: 0/24 (0·0%) - Sometimes: 4/24 (16·7%) - Rarely/never: 19/24 (79·2%) |
| **4. Organizational/logistical constraints**  To what extent do local healthcare organizational factors (e.g., difficulty securing hospital day-care slots for infusions, limited nursing resources, administrative hurdles) affect treatment choices in your center? | - Major influence: 4/24 (16·7%) - Moderate influence: 9/24 (37·5%) - Minor influence: 9/24 (37·5%) - No influence: 2/24 (8·3%) |
| **5. Role of clinical experience**  When choosing a treatment, how much weight do you place on your own clinical experience compared with guideline recommendations and published evidence? | - Much greater weight on personal experience: 0/24 (0%) - Balanced personal experience and guidelines: 23/24 (95·8%) - More weight on guidelines/evidence: 1/24 (4·2%) - Almost exclusively on guidelines/evidence: 0/24 (0%) |
| **6. Patient-related factors**  How influential are the following patient-specific factors in guiding therapeutic decisions in your center: |  |
| Overall | - Major influence: 1/24 (4·2%) - Moderate influence: 16/24 (66·7%) - Minor influence: 6/24 (25·0%) - No influence: 1/24 (4·2%) |
| Acute-onset CIDP | - Major influence: 9/24 (37·5%) - Moderate influence: 12/24 (50·0%) - Minor influence: 3/24 (12·5%) - No influence: 0/24 (0·0%) |
| Severe functional impairment | - Major influence: 10/24 (41·7%) - Moderate influence: 12/24 (50·0%) - Minor influence: 2/24 (8·3%) - No influence: 0/24 (0·0%) |
| Female gender | - Major influence: 0/24 (0·0%) - Moderate influence: 1/24 (4·2%) - Minor influence: 15/24 (62·5%) - No influence: 8/24 (33·3%) |
| Pediatric onset | - Major influence: 8/24 (33·3%) - Moderate influence: 10/24 (41·7%) - Minor influence: 3/24 (12·5%) - No influence: 3/24 (12·5%) |
| Pure-motor CIDP | - Major influence: 9/24 (37·5%) - Moderate influence: 10/24 (41·7%) - Minor influence: 4/24 (16·7%) - No influence: 1/24 (4·2%) |
| History of previous thrombosis | - Major influence: 7/24 (29·2%) - Moderate influence: 16/24 (66·7%) - Minor influence: 1/24 (4·2%) - No influence: 0/24 (0·0%) |
| Diagnosis of chronic kidney disease | - Major influence: 6/24 (25·0%) - Moderate influence: 13/24 (54·2%) - Minor influence: 5/24 (20·8%) - No influence: 0/24 (0·0%) |
| Diagnosis of diabetes mellitus | - Major influence: 4/24 (16·7%) - Moderate influence: 11/24 (45·8%) - Minor influence: 8/24 (33·3%) - No influence: 1/24 (4·2%) |
| Diagnosis of arterial hypertension | - Major influence: 0/24 (0·0%) - Moderate influence: 11/24 (45·8%) - Minor influence: 10/24 (41·7%) - No influence: 3/24 (12·5%) |
| History of previous gastritis and other gastric diseases | - Major influence: 1/24 (4·2%) - Moderate influence: 9/24 (37·5%) - Minor influence: 12/24 (50·0%) - No influence: 2/24 (8·3%) |
| IgM monoclonal gammopathy without anti-MAG antibodies | - Major influence: 5/24 (20·8%) - Moderate influence: 12/24 (50·0%) - Minor influence: 6/24 (25·0%) - No influence: 1/24 (4·2%) |
| **7.** **Patient preferences**  How much do patients’ preferences (e.g., home vs hospital therapy, oral vs infusion treatment, lifestyle considerations) influence choice of therapy in your center? | - Major influence: 1/24 (4·2%) - Moderate influence: 4/24 (16·7%) - Minor influence: 16/24 (66·7%) - No influence: 3/24 (12·5%) |
| **8.** **Center-level practices**  To what extent do treatment choices at your center are influenced by a shared “center approach” (e.g., local consensus, traditions, training background), as opposed to purely individual decisions? | - Strong influence of center-level practices: 2/24 (8·3%) - Moderate influence: 11/24 (45·8%) - Limited influence: 10/24 (41·7%) - No influence, decisions are always individualized: 1/24 (4·2%) |
| **9. Switching practices**  In your center, what are the most common reasons patients are switched from one treatment to another (e.g., IVIg → SCIg, IVIg → CS)? (multiple answers allowed) | - Lack of efficacy: 19/24 (79·2%) - Adverse events: 14/24 (58·3%) - Patient preference/convenience: 17/24 (70·8%) - Healthcare/economic reasons: 9/24 (37·5%) |
| **10. Barriers to guideline implementation**  In your opinion, what are the main barriers to fully implementing guideline recommendations at your center? (multiple answers allowed) | - Drug availability or reimbursement restrictions: 8/24 (33·3%) - Administrative or organizational limitations: 15/24 (62·5%) - Limited evidence or lack of clarity in guidelines: 5/24 (20·8%) - Local practice preferences or expertise: 6/24 (25·0%) |
