# Supplementary material for: Inter-centre heterogeneity, temporal evolution, and factors associated with treatment selection and outcomes in chronic inflammatory demyelinating polyradiculoneuropathy: a multicentre, combined prospective and retrospective observational study
Source: eClinicalMedicine. 2026 Jun 23;97:104031. doi: 10.1016/j.eclinm.2026.104031 (PMC13316210; doi:10.1016/j.eclinm.2026.104031)
Supplement: Supplementary Table S2 [file mmc2.docx]

# **Table S2 – Analysis of excluded patients**

LEGEND: CIDP= chronic inflammatory demyelinating polyradiculoneuropathy

|  | **Included**  **N=653** | **Excluded**  **N=122** | **p-value** |
| --- | --- | --- | --- |
| **Demographic** |  |  |  |
| Female gender, n (%) | 229/653 (35·1%) | 43/122 (35·2%) | 0·970 |
| Age at onset, years (SD) | 55·4 (14·4) | 49·6 (17·3) | <0·001 |
| Treatment delay, months (SD) | 38·37 (69·77) | 22·06 (38·93) | 0·010 |
| **CIDP phenotype** |  |  | 0·326 |
| Typical CIDP | 499 (76·4)% | 87 (71·3%) |  |
| Distal CIDP | 47 (7·2%) | 15 (12·3%) |  |
| Multifocal CIDP | 37 (5·7%) | 6 (4·9%) |  |
| Pure motor CIDP | 32 (4·9%) | 5 (4-1%) |  |
| Pure sensory CIDP | 27 (4·1%) | 8 (6·6%) |  |
| Autoimmune (para)nodopathy | 11 (1·7%) | 1 (0·8%) |  |
| **Treatment history** |  |  |  |
| Induction response, n/treatment data available | 492 (75·3%) | 36/53 (67-8%) | 0·232 |
| Minimal residual disability at last follow-up (INCAT 0–1), n/clinical data available | 235 (36·3%) | 48/112 (42·9%) | 0·183 |
