# Supplementary material for: Inter-centre heterogeneity, temporal evolution, and factors associated with treatment selection and outcomes in chronic inflammatory demyelinating polyradiculoneuropathy: a multicentre, combined prospective and retrospective observational study
Source: eClinicalMedicine. 2026 Jun 23;97:104031. doi: 10.1016/j.eclinm.2026.104031 (PMC13316210; doi:10.1016/j.eclinm.2026.104031)
Supplement: Supplementary Table S3 [file mmc3.docx]

# **Table S3 – Time evolution of diagnostic delay**

|  | **Diagnostic delay, median (IQR), months** | **p-value** |
| --- | --- | --- |
| **Time frame** |  | 0·371 |
| Before 2005 | 11·7 (1·8 - 48·5) |  |
| 2006-2010 | 11·9 (2·5 - 29·0) |  |
| 2011-2015 | 8·7 (3·4 - 46·4) |  |
| 2016-2020 | 8·7 (3·3 - 27·4) |  |
| 2021 - 2025 | 11·8 (6·0 - 43·7) |  |
