# Supplementary material for: Inter-centre heterogeneity, temporal evolution, and factors associated with treatment selection and outcomes in chronic inflammatory demyelinating polyradiculoneuropathy: a multicentre, combined prospective and retrospective observational study
Source: eClinicalMedicine. 2026 Jun 23;97:104031. doi: 10.1016/j.eclinm.2026.104031 (PMC13316210; doi:10.1016/j.eclinm.2026.104031)
Supplement: Supplementary Table S4 [file mmc4.docx]

# **Table S4 – Multinomial regression model of factors associated with the selection of induction therapy**

LEGEND: CIDP= chronic inflammatory demyelinating polyradiculoneuropathy; INCAT= inflammatory neuropathy cause and treatment score; MRC sum score= Medical Research Council sum score

|  | Model 1 | | model 2 | |
| --- | --- | --- | --- | --- |
|  | F value | p-value | F-value | p-value |
| Survey responses |  |  |  |  |
| Guideline influence |  |  | 1·33 | 0·2509 |
| Patient preferences |  |  | 1·26 | 0·2812 |
| Center-level practices |  |  | 1·72 | 0·1293 |
| Economic considerations |  |  | 2·03 | 0·0738 |
| Drug availability |  |  | 0·86 | 0·5046 |
| Organizational/logistical costraints |  |  | 2·55 | 0·0273 |
| Clinical picture and comorbidities |  |  | 3·39 | 0·0051 |
| Acute onset | 3·23 | 0·0071 | 4·59 | 0·0004 |
| Time period | 1·26 | 0·2007 | 1·28 | 0·1871 |
| Diabetes mellitus | 3·41 | 0·0048 | 3·64 | 0·0031 |
| Pure-motor CIDP | 2·03 | 0·0734 | 1·78 | 0·1148 |
| MRC sum score | 1·63 | 0·1508 | 2·19 | 0·0539 |
| IgM monoclonal gammopathy | 2·32 | 0·0422 | 2·14 | 0·0596 |
| Previous thrombosis | 1·06 | 0·3831 | 0·94 | 0·4531 |
| INCAT score | 0·94 | 0·4539 | 1·21 | 0·3042 |
