# Supplementary material for: Inter-centre heterogeneity, temporal evolution, and factors associated with treatment selection and outcomes in chronic inflammatory demyelinating polyradiculoneuropathy: a multicentre, combined prospective and retrospective observational study
Source: eClinicalMedicine. 2026 Jun 23;97:104031. doi: 10.1016/j.eclinm.2026.104031 (PMC13316210; doi:10.1016/j.eclinm.2026.104031)
Supplement: Supplementary Table S5 [file mmc5.docx]

**Table S5 – Odds-Ratios with 95% Confidence Intervals of multinomial regression models**

LEGEND: IVIg= intravenous immunoglobulins, CS= corticosteroids, PE= plasma exchange, IS= immunosuppressants, CIDP= chronic inflammatory demyelinating polyradiculoneuropathy; INCAT= inflammatory neuropathy cause and treatment score; MRC sum score= Medical Research Council sum score.

|  |  | Model 1 |  | model 2 |
| --- | --- | --- | --- | --- |
|  | Induction therapy | OR (95% CI) |  | OR (95% CI) |
| Acute onset | CS | 0,55 (0,29 - 1,06) |  | 0,57 (0,3 - 1,11) |
|  | IVIg + CS | 0,99 (0,39 - 2,47) |  | 1,06 (0,42 - 2,71) |
|  | PE | 5,91 (1,88 - 18,54) |  | 13,15 (3,73 - 46,38) |
|  | IS | 2,01 (0,3 - 13,52) |  | 1,8 (0,26 - 12,24) |
|  | SCIg | 0,02 (0 - 813,33) |  | 0,01 (0 - 885,75) |
| Time period 2 vs 1 | CS | 0,43 (0,22 - 0,85) |  | 0,45 (0,23 - 0,88) |
|  | IVIg + CS | 1,27 (0,4 - 4) |  | 1,22 (0,38 - 3,87) |
|  | PE | 0,65 (0,16 - 2,71) |  | 0,46 (0,1 - 2,15) |
|  | IS | 0,71 (0 - 182300000) |  | 0,68 (0 - 296677) |
|  | SCIg | 0,34 (0,07 - 1,64) |  | 0,4 (0,08 - 2,04) |
| Time period 3 vs 1 | CS | 0,56 (0,32 - 0,98) |  | 0,59 (0,33 - 1,03) |
|  | IVIg + CS | 1,5 (0,55 - 4,11) |  | 1,56 (0,56 - 4,32) |
|  | PE | 0,39 (0,1 - 1,5) |  | 0,25 (0,06 - 1,04) |
|  | IS | 57,74 (0 - 57369591) |  | 32,98 (0 - 539488) |
|  | SCIg | 0,04 (0 - 0,39) |  | 0,05 (0,01 - 0,48) |
| Time period 4 vs 1 | CS | 0,55 (0,3 - 0,99) |  | 0,53 (0,29 - 0,97) |
|  | IVIg + CS | 0,76 (0,24 - 2,34) |  | 0,8 (0,26 - 2,49) |
|  | PE | 0,17 (0,03 - 1,05) |  | 0,1 (0,02 - 0,66) |
|  | IS | 94,13 (0 - 92891772) |  | 38,45 (0 - 631297) |
|  | SCIg | 0,17 (0,04 - 0,84) |  | 0,21 (0,04 - 1,02) |
| Time period 5 vs 1 | CS | 0,64 (0,27 - 1,51) |  | 0,65 (0,28 - 1,53) |
|  | IVIg + CS | 0,9 (0,19 - 4,22) |  | 0,99 (0,2 - 4,85) |
|  | PE | 0,02 (0 - 49,92) |  | 0,01 (0 - 32,61) |
|  | IS | 45,72 (0 - 53480375) |  | 19,92 (0 - 436835) |
|  | SCIg | 0,01 (0 - 752,62) |  | 0 (0 - 854,42) |
| Diabetes mellitus | CS | 0,3 (0,15 - 0,63) |  | 0,29 (0,14 - 0,61) |
|  | IVIg + CS | 0,32 (0,09 - 1,12) |  | 0,33 (0,1 - 1,16) |
|  | PE | 0,4 (0,05 - 3,33) |  | 0,51 (0,06 - 4,62) |
|  | IS | 1,06 (0,19 - 5,93) |  | 1,36 (0,25 - 7,42) |
|  | SCIg | 2,97 (0,74 - 11,97) |  | 3,51 (0,83 - 14,84) |
| Pure motor CIDP | CS | 0,2 (0,07 - 0,61) |  | 0,24 (0,08 - 0,7) |
|  | IVIg + CS | 0,92 (0,28 - 3,04) |  | 0,99 (0,3 - 3,29) |
|  | PE | 0,01 (0 - 403,54) |  | 0,02 (0 - 689,99) |
|  | IS | 0,01 (0 - 40,39) |  | 0,01 (0 - 55,82) |
|  | SCIg | 0,01 (0 - 5102,38) |  | 0,01 (0 - 7059,85) |
| MRC sum score | CS | 1,04 (1 - 1,08) |  | 1,04 (1 - 1,08) |
|  | IVIg + CS | 1,03 (0,97 - 1,09) |  | 1,03 (0,97 - 1,1) |
|  | PE | 0,96 (0,89 - 1,04) |  | 0,98 (0,9 - 1,06) |
|  | IS | 0,93 (0,82 - 1,05) |  | 0,86 (0,74 - 0,99) |
|  | SCIg | 1,05 (0,93 - 1,19) |  | 1,07 (0,94 - 1,22) |
| IgM monoclonal gammopathy | CS | 3,08 (1,15 - 8,24) |  | 3,2 (1,2 - 8,55) |
|  | IVIg + CS | 1,71 (0,39 - 7,46) |  | 1,83 (0,41 - 8,15) |
|  | PE | 13,6 (2,6 - 71,04) |  | 12,33 (2,23 - 68,2) |
|  | IS | 0,08 (0 - 10030000000) |  | 0,4 (0 - 150821) |
|  | SCIg | 5,55 (0,49 - 63,53) |  | 5,11 (0,44 - 59,09) |
| Previous thrombosis | CS | 4,53 (1,08 - 19,12) |  | 4,13 (0,96 - 17,73) |
|  | IVIg + CS | 0,02 (0 - 168453) |  | 0,01 (0 - 824232) |
|  | PE | 4,52 (0,34 - 60,78) |  | 5,99 (0,41 - 87,59) |
|  | IS | 0,1 (0 - 2,461E+20) |  | 8,85 (0 - 5,982E+51) |
|  | SCIg | 8,39 (0,54 - 130,85) |  | 7,15 (0,46 - 112,27) |
| INCAT score | CS | 1,05 (0,92 - 1,19) |  | 1,06 (0,93 - 1,2) |
|  | IVIg + CS | 1,17 (0,97 - 1,41) |  | 1,18 (0,98 - 1,43) |
|  | PE | 1,22 (0,91 - 1,63) |  | 1,21 (0,88 - 1,64) |
|  | IS | 0,81 (0,48 - 1,35) |  | 0,67 (0,37 - 1,22) |
|  | SCIg | 1,07 (0,74 - 1,55) |  | 1,1 (0,74 - 1,65) |
| Guideline influence | CS |  |  | 1,29 (0,74 - 2,22) |
|  | IVIg + CS |  |  | 1,55 (0,67 - 3,61) |
|  | PE |  |  | 2,35 (0,51 - 10,78) |
|  | IS |  |  | 0,01 (0 - 1,69) |
|  | SCIg |  |  | 0,48 (0,08 - 2,92) |
| Patient preferences influence | CS |  |  | 0,92 (0,61 - 1,38) |
|  | IVIg + CS |  |  | 1,1 (0,6 - 2,02) |
|  | PE |  |  | 0,23 (0,03 - 1,8) |
|  | IS |  |  | 13,92 (0,54 - 361,62) |
|  | SCIg |  |  | 0,33 (0,06 - 1,91) |
| Center level practicies influence | CS |  |  | 0,69 (0,48 - 1,01) |
|  | IVIg + CS |  |  | 0,77 (0,44 - 1,33) |
|  | PE |  |  | 1,13 (0,41 - 3,13) |
|  | IS |  |  | 77,27 (0,76 - 7820,33) |
|  | SCIg |  |  | 2 (0,4 - 9,97) |
| Economic considerations | CS |  |  | 0,9 (0,62 - 1,3) |
|  | IVIg + CS |  |  | 0,67 (0,37 - 1,19) |
|  | PE |  |  | 0,5 (0,16 - 1,55) |
|  | IS |  |  | 18,3 (1,69 - 197,73) |
|  | SCIg |  |  | 1,63 (0,51 - 5,24) |
| Drug availability influence | CS |  |  | 0,87 (0,67 - 1,15) |
|  | IVIg + CS |  |  | 1,28 (0,83 - 2) |
|  | PE |  |  | 1,08 (0,55 - 2,1) |
|  | IS |  |  | 0,46 (0,11 - 1,9) |
|  | SCIg |  |  | 0,71 (0,3 - 1,68) |
| Organizational/logistical influence | CS |  |  | 1,4 (0,99 - 2) |
|  | IVIg + CS |  |  | 1,32 (0,74 - 2,36) |
|  | PE |  |  | 0,65 (0,26 - 1,62) |
|  | IS |  |  | 26,26 (2,17 - 317,43) |
|  | SCIg |  |  | 0,64 (0,25 - 1,64) |
| Clinical picture/comorbidities influence | CS |  |  | 1,88 (1,17 - 3,02) |
|  | IVIg + CS |  |  | 2,58 (1,21 - 5,5) |
|  | PE |  |  | 0,79 (0,19 - 3,3) |
|  | IS |  |  | 0·02 (0 - 0·6) |
|  | SCIg |  |  | 0·99 (0·21 - 4·66) |
