# Supplementary material for: Inter-centre heterogeneity, temporal evolution, and factors associated with treatment selection and outcomes in chronic inflammatory demyelinating polyradiculoneuropathy: a multicentre, combined prospective and retrospective observational study
Source: eClinicalMedicine. 2026 Jun 23;97:104031. doi: 10.1016/j.eclinm.2026.104031 (PMC13316210; doi:10.1016/j.eclinm.2026.104031)
Supplement: Supplementary Table S6 [file mmc6.docx]

# **Table S6 – Determinants of IVIg dosage at maintenance**

|  | **Mean (SD) monthly g/kg or R value** | | **p-value** |
| --- | --- | --- | --- |
| **Demographic** |  |  |  |
| Age at onset | R= -0·009 | | 0·887 |
| Female gender | Female  1·04 (0·69) | Male  1·01 (0·66) | 0.606 |
| **CIDP features** |  |  |  |
| Possible CIDP on EFNS/PNS criteria | Yes  1·02 (0·68) | No  1·01 (0·62) | 0.952 |
| Disease history | Relapsing  1·04 (0·66) | Progressive  1·03 (0·69) | 0.869 |
| Acute onset | Yes  1·11 (0·73) | No  1·00 (0·66) | 0.581 |
| CIDP phenotype | Typical CIDP  0·99 (0·66) | Atypical CIDP  1·18 (0·73) | 0.126 |
| Pediatric onset | Yes  1·30 (0·93) | No  1·01 (0·65) | 0.500 |
| **Comorbidities** |  |  |  |
| Concurrent immune disease | Yes  1·14 (0·63) | No  1·02 (0·68) | 0.246 |
| Diabetes mellitus | Yes  0·96 (0·59) | No  1·03 (0·68) | 0.961 |
| Monoclonal gammopathy of unknown significance | Yes  0·94 (0·73) | No  1·03 (0·67) | 0.313 |
| **Outcome measures** |  |  |  |
| INCAT | R= 0·088 | | 0·176 |
| MRC | R= -0·081 | | 0·213 |
| RODS | R= -0·040 | | 0·536 |
