# Supplementary material for: Inter-centre heterogeneity, temporal evolution, and factors associated with treatment selection and outcomes in chronic inflammatory demyelinating polyradiculoneuropathy: a multicentre, combined prospective and retrospective observational study
Source: eClinicalMedicine. 2026 Jun 23;97:104031. doi: 10.1016/j.eclinm.2026.104031 (PMC13316210; doi:10.1016/j.eclinm.2026.104031)
Supplement: Supplementary Table S7 [file mmc7.docx]

# **Table S7 – Therapeutic response to immunosuppressants**

| **Indication** |  | **IVIg- or CS- sparing** | | |  | **Rescue therapy** |  |  |
| --- | --- | --- | --- | --- | --- | --- | --- | --- |
|  | **n** | **Suspended other treatments** | **Maintained other treatments** | **Worsened or resumed other treatments** |  | **Improved** |  | **Adverse events** |
| **Immunosuppressant** |  |  |  |  |  |  |  |  |
| Azathioprine | 81 | 24/57 (42·1%) | 28/57 (49·1%) | 19/57 (33·3%) |  | 8/24 (33·3%) |  | 15/81 (18·5%) |
| Rituximab | 27 | 6/10 (60·0%) | 1/10 (10·0%) | 3/10 (30·0%) |  | 10/15 (66·7%) |  | 9/27 (33·3%) |
| Cyclosporine | 11 | 2/6 (33·3%) | 1/6 (16·7%) | 3/3 (100·0%) |  | 2/4 (50·0%) |  | 3/11 (27·3%) |
| Methotrexate | 10 | 4/6 (66·7%) | 0/6 (0·0%) | 2/6 (33·3%) |  | 0/4 (0·0%) |  | 0/10 (0·0%) |
| Cyclophosphamide | 9 | 5/5 (100·0%) | 0/5 (0·0%) | 0/5 (0·0%) |  | 2/4 (50·0%) |  | 0/9 (0·0%) |
| Mycophenolate mofetil | 6 | 0/2 (0·0%) | 0/2 (0·0%) | 2/2 (100·0%) |  | 2/4 (50·0%) |  | 3/6 (50·0%) |
| Interferon-beta | 3 | 1/2 (50·0%) | 0/2 (0·0%) | 1/2 (50·0%) |  | 0/1 (0·0%) |  | 0/3 (0·0%) |
